# Randomized, controlled, multi‐center phase II study of postoperative enoxaparin treatment for venous thromboembolism prophylaxis in patients undergoing surgery for hepatobiliary‐pancreatic malignancies

**DOI:** 10.1002/ags3.12796

**Published:** 2024-03-28

**Authors:** Go Shinke, Yutaka Takeda, Yoshiaki Ohmura, Shogo Kobayashi, Hiroshi Wada, Osakuni Morimoto, Akira Tomokuni, Junzo Shimizu, Tadafumi Asaoka, Masahiro Tanemura, Takehiro Noda, Yuichiro Doki, Hidetoshi Eguchi

**Affiliations:** ^1^ Department of Surgery Kansai Rosai Hospital Amagasaki Hyogo Japan; ^2^ Department of Gastroenterological Surgery Osaka University Suita Osaka Japan; ^3^ Department of Surgery JCHO Osaka Hospital Osaka Japan; ^4^ Department of Gastroenterological Surgery Osaka General Medical Center Osaka Japan; ^5^ Department of Surgery Toyonaka Municipal Hospital Toyonaka Osaka Japan; ^6^ Department of Gastroenterological Surgery Osaka Police Hospital Osaka Japan; ^7^ Department of Surgery Rinku General Medical Center Izumisano Osaka Japan

**Keywords:** DVT, enoxaparin, hepatobiliary‐pancreatic malignancy, PTE, VTE prophylaxis

## Abstract

**Purpose:**

Postoperative venous thromboembolism (VTE) risk is pronounced after abdominal cancer surgery. Enoxaparin shows promise in preventing VTE in gastrointestinal, gynecological, and urological cancers, but its application after surgery for hepatobiliary‐pancreatic malignancy has been under‐evaluated due to bleeding concerns. We confirmed the safety of enoxaparin administration in patients undergoing curative hepatobiliary‐pancreatic surgery for malignancies in a prospective, multi‐center, phase I study.

**Methods:**

The study was conducted from April 2015 to May 2021 across eight specialized centers. Patients (*n* = 262) were randomized to enoxaparin prophylaxis given postoperatively for 8 days (*n* = 131) or control (*n* = 131). The primary endpoint was the efficacy in reducing VTE. Secondary endpoints examined safety.

**Results:**

The full analysis set included 259 patients (131 control, 129 enoxaparin). The per‐protocol population included 233 patients (117 control, 116 enoxaparin). Most cases were hepatic malignancies (111 control, 111 enoxaparin). The median administration duration of enoxaparin was 7 days, with 92% receiving 4000 units/day. Despite a reduction in the relative risk (RR) of VTE due to postoperative enoxaparin administration, the results were not significant (control: four cases, 3.4% vs. treatment: two cases, 1.7%; RR 0.50, 95% CI 0.09–2.70; *p* = 0.6834). No significant difference was found in the incidence of bleeding events (control: five cases, 4.3% vs. treatment: five cases, 4.3%, RR 1.00, 95% CI 0.53–1.89; *p* = 1.0000).

**Conclusions:**

The perioperative administration of enoxaparin in hepatobiliary‐pancreatic malignancies is feasible and safe. However, further case accumulation and investigation are necessary to assess its potential in reducing the occurrence of VTE.

## Graphical abstract

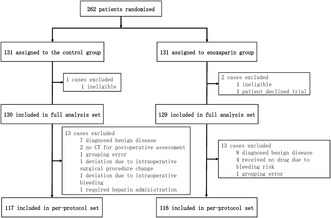

## 1 INTRODUCTION

Venous thromboembolism (VTE) can be classified as deep venous thrombosis (DVT) or pulmonary thromboembolism (PTE). PTE is a condition caused by the embolization of a thrombus formed in the veins or heart, which can result in potentially fatal outcomes due to obstruction of the pulmonary vessels. More than 90% of the embolic sources of PTE originate from the lower limbs or pelvic veins, making postoperative VTE prevention necessary. VTE is more likely to occur after abdominal cancer surgery.^1^ The guidelines of the American College of Chest Physicians (ACCP) recommend using heparin, low‐molecular‐weight heparin, or fondaparinux for moderate‐ to high‐risk surgeries in the field of general surgery.^2^ In Japan, because the incidence of postoperative VTE is comparable to the incidence in Western countries, VTE prevention is considered necessary.^3^ The “Guidelines for the Diagnosis, Treatment, and Prevention of Pulmonary Thromboembolism and Deep Vein Thrombosis (Revised in 2009)” recommend VTE prevention according to the ACCP guidelines.^4^ However, there is a lack of evidence, and target cases and methods of administration have not been established.

Enoxaparin is a low‐molecular‐weight heparin that has been shown to be effective in preventing VTE.^5^ In clinical studies in Japan, the efficacy of enoxaparin has been reported mainly for gastrointestinal, gynecological, and urological cancers.^6^ It is expected to have a lower risk of bleeding than heparin. Though patients undergoing surgery for hepatobiliary‐pancreatic malignancies are considered a high‐risk group for VTE development, there are also concerns regarding the bleeding risks associated with the use of VTE prophylactic agents. As a result, VTE prevention using medications has not been thoroughly implemented in this field. In domestic clinical trials of enoxaparin, which are indicated for VTE prevention after abdominal surgery in Japan, there have been only three cases of liver surgery and one case of pancreatic surgery, and the safety and efficacy were not fully evaluated.^6^

Through our prospective, multi‐center, phase I study on postoperative enoxaparin treatment in patients undergoing curative hepatobiliary‐pancreatic surgery for malignancies,^7^ we confirmed the safety of enoxaparin administration. The objective of the present study was to analyze the effectiveness of enoxaparin administration after surgery for hepatobiliary‐pancreatic malignancies.

## 2 METHODS

### 2.1 Patients

This randomized, controlled, multi‐center, phase II study was started in April 2015 and ended in May 2021, with participation from eight specialized centers. This trial was conducted as an intergroup cooperative study led by the Clinical Study Group of Osaka University, Hepato‐Biliary Pancreatic Group (CSGO‐HBP). Approval for this study was obtained from the Ethics Committee of the Graduate School of Medicine, Osaka University. This study was registered with the University Hospital Medical Information Network Clinical Trials Registry, an officially accepted registry by the International Committee of Medical Journal Editors (registration number UMIN000019368). Clinical data were collected from patient case report forms.

Patients of both sexes were eligible when they were scheduled for surgery for hepatobiliary‐pancreatic malignancy. The main inclusion criteria were age ≥40 years, Child–Pugh Grade A, and a life expectancy of at least 6 months. The exclusion criteria were a history of prior DVT; receiving estrogen or progesterone within 4 weeks prior to drug initiation in the study; systemic chemotherapy or radiation therapy received within 2 weeks before drug initiation in the study; peri‐operative use of anticoagulants and antiplatelets; active bleeding; severe renal failure, indicated by an estimated glomerular filtration rate (eGFR) < 30 mL/min; platelet count (PLT) < 75 000/mm; activated partial thromboplastin time (APTT) > 50 s; history of heparin‐induced thrombocytopenia; eye, spinal cord, or central nervous system surgery within 2 weeks prior to surgery; active peptic ulcer; cerebral hemorrhage; or endocarditis. Patients with a contraindication to anticoagulant prophylaxis and pregnant or lactating women were also excluded. These criteria are same as those of the phase I study.^7^

### 2.2 Randomization

Eligible patients were centrally registered at a nonprofit organization, the Supporting Center for Clinical Research and Education (SCCRE), Osaka, Japan. Block randomization was performed by a computer‐generated random number list prepared by staff at SCCRE with no clinical involvement in the trial. Upon registration, the allocation of subcutaneous enoxaparin injection for prophylaxis was randomly assigned by the SCCRE. For the random allocation, a minimization method with adjustment factors was used to ensure no significant imbalances were present in facility, gender, age, or disease. Physical therapy for VTE prevention, such as elastic bandages, elastic stockings, and intermittent pneumatic compression, was performed intraoperatively for participants in both the control group and enoxaparin group. Enoxaparin was administered to the treatment group for a total of 8 days, from the second day after surgery until the ninth day.

### 2.3 Enoxaparin treatment

The following treatment method is being carried out in the same way as the method adopted in the phase I study.^7^ Before subcutaneous administration of the study medication, investigators checked the laboratory tests to ensure PLT ≥ 75 000/mm and APTT < 50 s at 1 and 2 days after surgery. After confirming no active bleeding, enoxaparin was initiated 2 days post‐operation and continued for 8 days. When surgeons considered that the risk of postoperative bleeding was high, pharmacological thromboprophylaxis could be delayed. We previously demonstrated in the phase I study that 20 mg enoxaparin administered twice daily is safe for VTE prophylaxis in select Japanese patients undergoing curative hepatobiliary‐pancreatic surgery for malignancies. Patients received a subcutaneous injection of enoxaparin twice daily for 8 days when renal function was normal (eGFR >50 mL/min). Because renal impairment reduces drug clearance, leading to an increase in exposure to enoxaparin sodium, patients with moderate renal impairment (eGFR between 50 and 30 mL/min) received 20 mg once daily for 8 days. These patients also received mechanical thromboprophylaxis with knee‐high, graduated compression stockings. The morning enoxaparin dose was skipped on the day of epidural catheter removal because the epidural catheter must be removed at least 10–12 h after the last enoxaparin dose and the next enoxaparin dose given at least 2 h after epidural catheter removal. Enoxaparin administration was terminated if the patient showed major or minor bleeding or abnormal PLT counts.

### 2.4 Primary endpoints

The purpose of this study was to evaluate the efficacy of subcutaneous enoxaparin in reducing the frequency of symptomatic and asymptomatic VTE, for the prevention of postoperative VTE in patients with hepato‐biliary malignancy, in an open label study. VTE was evaluated on postoperative days 7–11 by contrast CT of the thorax, abdomen, and lower extremities in all participants. VTE was assessed from the initiation of enoxaparin administration until 30 days after surgery and classified as DVT or PTE. Thrombus location was assessed and included the subclavian veins, brachiocephalic vein, superior vena cava, pulmonary artery, pulmonary artery main branches, pulmonary artery secondary branches, inferior vena cava, iliac veins, external iliac veins, internal iliac veins, great saphenous veins, deep femoral veins, popliteal veins, anterior tibial veins, posterior tibial veins, fibular veins, and peroneal veins.

### 2.5 Secondary endpoints

The safety of enoxaparin in patients undergoing hepatobiliary‐pancreatic surgery for malignancies was determined based on the incidence of bleeding events from the initiation of enoxaparin administration until postoperative day 30. Major bleeding was defined according to the definitions described in a previous Japanese study: bleeding leading to death; retroperitoneal bleeding, intracranial bleeding, bleeding in vital organs (e.g., eyeball, adrenal gland, pericardium, spine); bleeding or hematoma requiring surgical intervention at the surgical site; bleeding associated with a decrease in hemoglobin (Hb) ≥ 2 g/dL within 48 h after bleeding, compared to the pre‐bleeding value; bleeding requiring transfusion of ≥800 mL of whole blood within 48 h after bleeding, or a transfusion of red blood cells derived from ≥800 mL of whole blood.

### 2.6 Sample size

The VTE incidence rate 12 days after abdominal surgery in Japan is 24.3%,^3^ including asymptomatic cases. The VTE incidence rate in a mechanical prophylaxis group during the development of enoxaparin was reported to be 19.4% and in a mechanical prophylaxis group during the development of fondaparinux 17.6%.^6^ Therefore, the VTE incidence rate in the non‐administration group was assumed to be 20%. Considering that the incidence rate of symptomatic VTE in patients receiving anticoagulant therapy after liver resection was reported to be 2.2% compared to 6.3% in the non‐implementation group,^6^ the VTE incidence rate in the administration group was set at 7% to assume a reduction of approximately one‐third, including asymptomatic VTE. Assuming VTE incidence rates of 20% in the non‐administration group and 7% in the administration group, and conducting a two‐sided chi‐squared test with *α* = 0.05 and *β* = 0.2, a total of 108 cases (216 cases total) were required in each group. Considering non‐administration and exclusion cases, the required number of cases was set at 260. The total number of surgeries for malignant tumors in the hepatobiliary‐pancreatic region at the participating facilities exceeds 200 cases annually. However, patients who cannot undergo contrast‐enhanced CT due to contrast agent allergies were excluded. In addition, patients with ongoing bleeding from drains or other factors and cases in which postoperative examination did not meet the hemostatic function criteria were not administered enoxaparin. Cases diagnosed as malignant tumors before surgery but diagnosed as benign tumors by postoperative pathology were also excluded from the analysis. Based on these considerations, the registration period was set at 5 years.

### 2.7 Statistical analysis

Clinical data are expressed as the mean ± standard deviation or median. The primary endpoint and secondary endpoint were based on the full analysis set and per‐protocol set. The chi‐squared test and Fisher's exact test were used to compare categorical variables when appropriate. All analyses were carried out in the JMP 16 software program (SAS Institute, Cary, NC, USA). Significance was defined as *p* = 0.05.

## 3 RESULTS

**FIGURE 1:**
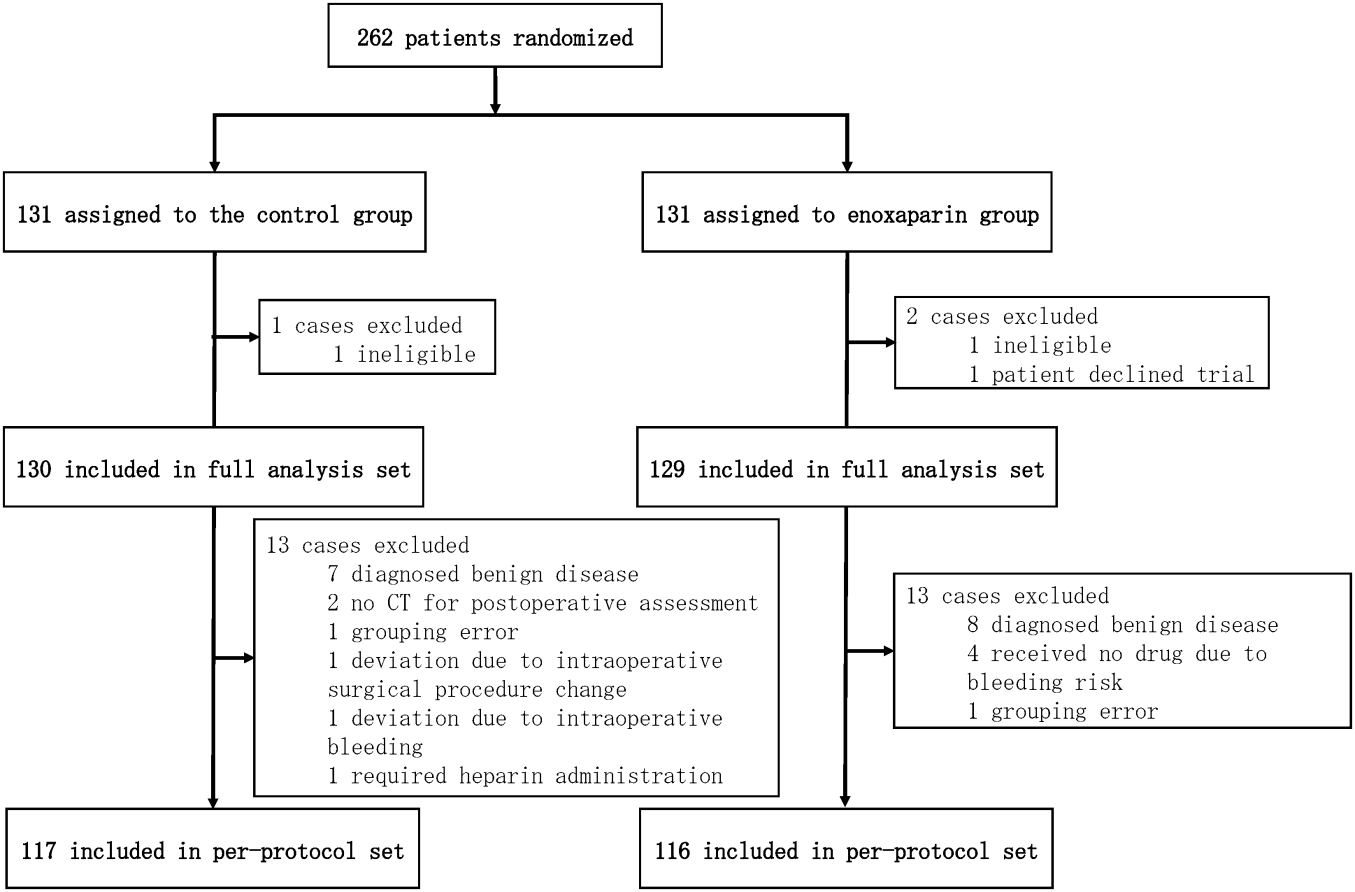
Disposition of patients randomly assigned to the control group or enoxaparin group.

Between April 2015 and May 2021, 262 patients were enrolled and randomly assigned to the control group (*n* = 131) or enoxaparin group (*n* = 131). The full analysis set comprised 259 patients (131 in the control group and 129 patients in the enoxaparin group) following the exclusion of three patients, including two patients (one in the control group and one in the enoxaparin group) found to be ineligible after randomization and one patient who declined participation after randomization. The per‐protocol population comprised 233 patients (117 in the control group and 116 in the enoxaparin group) following the exclusion of 26 patients, including 15 patients (seven in the control group and eight in the enoxaparin group) who were diagnosed with benign disease by histopathology, four patients who did not receive enoxaparin for bleeding risk, two patients in the control group who were not examined by CT for postoperative assessment, two patients (one in the control group and one in the enoxaparin group) who had a mistake in their group classification, one patient in the control group who deviated due to an intraoperative change in surgical procedure, one patient in the control group who deviated due to intraoperative bleeding, and one patient in the control group who required heparin administration (Figure 1).

Baseline characteristics were well balanced between the two groups. Most cases were hepatic malignancies (111 patients in each group). The most common surgical procedure performed was hepatectomy, including 112 patients (88.2%) in the control group and 116 patients (89.9%) in the enoxaparin group. We found no significant difference in operation time between the two groups (348.1 ± 148.7 vs. 351.0 ± 148.2, *p* = 0.8757). In the enoxaparin group, the median duration of enoxaparin administration was 7 days (range: 0–10 days), and the dose administered was 4000 units/day in 119 patients (92%). The remaining patients received a dose of 2000 units/day.

We found no significant difference in the incidence of VTE between the two groups [four cases, 3.2% vs. three cases, 2.3%; relative risk (RR) 0.74, 95% confidence interval (CI) 0.17–3.23; *p* = 0.7211; Table 1]. When analyzing the incidences of PTE and DVT separately, no significant differences were found (one case, 0.8% vs. three cases, 1.6%; RR 1.97, 95% CI 0.18–21.44; *p* = 1.0000; and three cases, 2.4% vs. two cases, 1.6%; RR 0.66, 95% CI 0.11–3.86; *p* = 0.6826). The incidence of bleeding events also did not significantly differ (five cases, 3.9% vs. five cases, 3.9%; RR 0.98, 95% CI 0.29–3.32; *p* = 1.0000). When analyzing major bleeding and minor bleeding events separately, no significant differences were found (one case, 0.8% vs. zero cases; *p* = 0.4961; four cases, 3.2% vs. five cases, 3.9%; RR 1.23, 95% CI 0.34–4.48; *p* = 1.0000).

**TABLE 1:** Full analysis set.

		Control group	Enoxaparin group	RR (95% Cl)	*p*‐value
		*n* = 130	*n* = 129		
Age, years	Mean ± SD	69.2 ± 9.4	68.7 ± 9.9		0.6992
Gender, *n*	Male/female	86/44	88/41		0.7916
Height, cm	Mean ± SD	160.8 ± 9.1	161.9 ± 8.1		0.3390
Weight, kg	Mean ± SD	61.2 ± 12.3	61.7 ± 11.9		0.7170
BMI, kg/m^2^	Mean ± SD	23.5 ± 3.7	23.5 ± 3.8		0.9289
Disease, *n* (%)	Hepatic malignancies	111 (88.1%)	111 (86.7%)		0.5982
	Biliary malignancies	3 (2.4%)	6 (4.7%)		
	Pancreatic malignancies	12 (9.5%)	11 (8.6%)		
Administration period of enoxaparin, days	Median (Min–Max)	0 (0–8)	7 (0–10)		
Dose of enoxaparin, *n*	2000/4000 IU/day	0/1	4/119		
Operative time, min	Mean ± SD	348.1 ± 148.7	351.0 ± 148.2		0.8757
Surgical procedures, *n* (%)	DP	12 (9.5%)	10 (7.8%)		0.8885
	LR	112 (88.2%)	116 (89.9%)		
	Others	3 (2.4%)	3 (2.3%)		
Outcome, *n* (%)					
VTE		4 (3.2%)	3 (2.3%)	0.74 (0.17–3.23)	0.7211
PTE		1 (0.8%)	2 (1.6%)	1.97 (0.18–21.44)	1.0000
DVT		3 (2.4%)	2 (1.6%)	0.66 (0.11–3.86)	0.6826
Bleeding events		5 (3.9%)	5 (3.9%)	0.98 (0.29–3.32)	1.0000
Major bleeding		1 (0.8%)	0	0	0.4961
Minor bleeding		4 (3.2%)	5 (3.9%)	1.23 (0.34–4.48)	1.0000

Abbreviations: DP, distal pancreatectomy; DVT, deep venous thrombosis; LR, liver resection; PTE, pulmonary thromboembolism; RR, relative risk; SD, standard deviation; VTE, venous thromboembolism.

In the per‐protocol set (Table 2), we found no significant difference in the incidence of VTE between the two groups (four cases, 3.4% vs. two cases, 1.7%; RR 0.50, 95% CI 0.09–2.70; *p* = 0.6834). When analyzing the incidences of PTE and DVT separately, no significant differences were found (one case, 0.9% vs. one case, 0.9%; RR 1.01, 95% CI 0.06–15.93; *p* = 1.0000; and three cases, 2.6% vs. two cases, 1.7%; RR 0.67, 95% CI 0.11–3.95; *p* = 1.0000). The incidence of bleeding events did not significantly differ (five cases, 4.3% vs. five cases, 4.3%; RR 1.00, 95% CI 0.53–1.89; *p* = 1.0000). When analyzing major bleeding and minor bleeding events separately, no significant differences were found (one case, 0.9% vs. zero cases, *p* = 1.0000; and four cases, 3.4% vs. five cases, 4.3%; RR 1.26, 95% CI 0.35–4.58; *p* = 0.7485).

**TABLE 2:** Per‐protocol set.

		Control group	Enoxaparin group	RR (95% Cl)	*p*‐value
		*n* = 117	*n* = 116		
Age, years	Mean ± SD	69.0 ± 9.6	68.7 ± 10.1		0.8314
Gender, *n*	Male/female	78/39	83/33		0.4790
Height, cm	Mean ± SD	160.8 ± 8.7	162.1 ± 8.2		0.2690
Weight, kg	Mean ± SD	60.7 ± 11.7	62.3 ± 12.0		0.2831
BMI, kg/m^2^	Mean ± SD	23.3 ± 3.5	23.7 ± 3.9		0.5011
Disease, *n* (%)	Hepatic malignancies	102 (87.2%)	104 (89.7%)		0.2214
	Biliary malignancies	3 (2.7%)	6 (5.2%)		
	Pancreatic malignancies	12 (0.3%)	6 (5.2%)		
Administration period of enoxaparin, days	Median (Min–Max)		7 (1–10)		
Dose of enoxaparin, *n*	2000/4000 IU/day		3/113		
Operative time, min	Mean ± SD	344.8 ± 147.8	346.6 ± 144.7		0.9275
Surgical procedures, *n* (%)	DP	11 (9.4%)	6 (5.2%)		0.0955
	LR	103 (88.0%)	110 (94.8%)		
	Others	3 (2.6%)	0		
Outcome, *n* (%)					
VTE		4 (3.4%)	2 (1.7%)	0.50 (0.09–2.70)	0.6834
PTE		1 (0.9%)	1 (0.9%)	1.01 (0.06–15.93)	1.0000
DVT		3 (2.6%)	2 (1.7%)	0.67 (0.11–3.95)	1.0000
Bleeding events, *n* (%)		5 (4.3%)	5 (4.3%)	1.00 (0.53–1.89)	1.0000
Major bleeding		1 (0.9%)	0	0	1.0000
Minor bleeding		4 (3.4%)	5 (4.3%)	1.26 (0.35–4.58)	0.7485

Abbreviations: DP, distal pancreatectomy; DVT, deep venous thrombosis; LR, liver resection; PTE, pulmonary thromboembolism; RR, relative risk; SD, standard deviation; VTE, venous thromboembolism.

Further analysis was performed on the details of VTE cases in Table 3. A total of seven cases (four in the control group and three in the enoxaparin group) were identified. One case was benign according to postoperative pathological examination. In the enoxaparin group, all cases received a dose of 4000 units/day, and the duration of administration was more than 6 days. The diseases in the VTE cases comprised four cases of colorectal cancer liver metastasis (CRC meta, *n* = 3 controls, *n* = 1 enoxaparin‐treated patient), one case of pancreatic ductal adenocarcinoma (PDAC, *n* = 1 control), one case of hepatocellular carcinoma (HCC, enoxaparin group), and one case of mucinous cyst adenoma (MCA, enoxaparin group). Four cases had laparoscopic surgery (*n* = 2 controls, *n* = 2 enoxaparin‐treated patients) and three cases underwent open surgery (*n* = 2 controls, *n* = 1 enoxaparin‐treated patient). Surgical procedures for malignant liver tumors were all partial liver resection, whereas surgeries for pancreatic tumors involved distal pancreatectomy with splenectomy. No significant differences were observed in terms of intraoperative blood loss, operation time, or postoperative hospital stay in the cases of VTE occurrence in the enoxaparin group. One case of VTE occurrence in the enoxaparin group had a history of diabetes. Another case had a history of multiple surgeries, including uterine fibroid surgery, appendectomy, cholecystectomy, and thyroid cancer surgery.

**TABLE 3:** VTE cases.

Group	Control group	Control group	Control group	Control group	Enoxaparin group	Enoxaparin group	Enoxaparin group
Benign/malignancy	Malignancy	Malignancy	Malignancy	Malignancy	Malignancy	Malignancy	Benign
Administration dose, IU	—	—	—	—	4000	4000	4000
Administration period, days	—	—	—	—	8	8	6
VTE							
PTE	No	No	Yes	No	No	Yes	Yes
DVT	Yes	Yes	No	Yes	Yes	Yes	No
Onset site of DVT	IJV, PoV, PTA	IJV	—	SoV	SoV	SoV	—
Postoperative days to DVT onset	7	7	7	8	8	10	7
Bleeding events	No	No	No	No	No	No	No
Sex	Female	Female	Male	Female	Female	Male	Female
Age, years	75	76	68	49	57	70	51
Height, cm	145.8	146.4	169.7	160.5	159	163	161
Weight, kg	44.1	47.2	64.1	67.8	57	58	54.6
BMI, kg/m^2^	20.7	22	22.3	26.3	22.5	21.8	21.1
Disease detail	CRCmeta	CRCmeta	PDAC	CRCmeta	CRCmeta	HCC	MCA
Open/ laparoscopic surgery	Lap.	Lap.	Open	Open	Open	Lap.	Lap.
Surgical procedure	LR	LR	DP	LR	LR	LR	DP
Surgical procedure details	Partial LR	Partial LR	DP, splenectomy	Partial LR	Partial LR	Partial LR	DP, splenectomy
Operative time, min	561	484	655	222	221	160	448
Intraoperative blood loss, mL	100	Small amount	1240	100	300	130	Small amount
Transfusion, yes/no	No	No	No	No	No	No	No
Postoperative hospital stays, days	9	9	24	17	13	13	8

Abbreviations: CRCmeta, colorectal carcinoma metastasis; DP, distal pancreatectomy; DVT, deep venous thrombosis; HCC, hepatocellular carcinoma; IJV, internal jugular vein; LR, liver resection; MCA, mucinous cystic adenoma; PDAC, pancreatic ductal adenocarcinoma; PoV, popliteal vein; PTA, posterior tibial vein; PTE, pulmonary thromboembolism; RR, relative risk; SoV, soleal vein; VTE, venous thromboembolism.

## 4 DISCUSSION

No randomized comparative studies have previously addressed perioperative VTE prophylaxis for surgery of hepatobiliary‐pancreatic malignancies. Though this study revealed a reduction in the RR of VTE due to postoperative enoxaparin administration, the results did not achieve significance, as the 95% CI crossed the threshold of 1. A secondary evaluation of the safety of perioperative enoxaparin administration for hepatobiliary‐pancreatic surgery included an increased sample size based on the results of a previous phase I study. These expanded results further contribute to the growing body of evidence supporting the safety of enoxaparin administration in hepatobiliary‐pancreatic surgery.

Postoperative enoxaparin administration for hepatobiliary‐pancreatic malignancies is expected to ensure safety and reduce the incidence of VTE. Though randomized comparative trials investigating the effectiveness of VTE prevention through enoxaparin administration are currently lacking, studies addressing thromboprophylaxis in the context of hepatobiliary‐pancreatic surgery have been reported,^8^, ^9^ with scattered reports on liver and pancreatic resections.^10^, ^11^, ^12^, ^13^ Because no randomized comparative trials on the effectiveness of enoxaparin administration for VTE prevention are available, this study, despite not yielding significant differences, is considered to provide important insights for future VTE prevention strategies in hepatobiliary‐pancreatic surgery for malignancies.

The underpowered nature of this study can be attributed to a discrepancy between the initially anticipated VTE incidence rates during study planning and the actual rates observed during the study. The VTE incidence rates in this study were low: 3.4% in the control group and 1.7% in the enoxaparin group, compared to the expected 20% for a non‐administration group and 7% for an administration group. The reasons for the significant deviation from the initially projected VTE incidence rates inferred from past studies could be attributed to various factors, such as faster postoperative recovery and mobilization in recent perioperative management, improved surgical techniques, and an increasing prevalence of laparoscopic surgery in the hepatobiliary‐pancreatic field, reflecting trends in other areas. In particular, Lancellotti et al. referred that a comparison between laparoscopic liver resection (LLR) and open LR (OLR) suggests a lower incidence of VTE in LLR.^14^ Furthermore, the relatively small number of pancreatic surgery cases in this study should be noted. Considering these factors, it is conceivable that the actual occurrence of VTE was lower than initially predicted. Accumulating more cases may provide a higher likelihood of achieving significant results in preventing VTE through enoxaparin administration.

Despite perioperative enoxaparin administration, cases of VTE were observed. As demonstrated in the results, diabetes mellitus, splenectomy, history of hysterectomy, or multiple prior general anesthesia surgeries led to the occurrence of VTE even with enoxaparin administration. These factors have been reported to increase the risk of VTE in previous studies.^15^, ^16^, ^17^, ^18^ Earlier studies discussing VTE risk factors commonly list factors related to the patient (such as being female, having a higher American Society of Anesthesiologists class, relying on a ventilator, experiencing breathing difficulties before surgery, having widespread cancer, undergoing chemotherapy within a month, and receiving red blood cells within 72 h before the operation), preoperative blood test results (such as low albumin levels, high bilirubin levels, elevated sodium levels, and low hematocrit levels), as well as the characteristics of the operation itself (such as the type of surgical procedure, whether it was an emergency, work relative value units, and the presence of infected or contaminated wounds).^19^ Gangireddy et al.^20^ highlighted urinary tract infection, acute renal failure, postoperative transfusion, perioperative myocardial infarction, and pneumonia as the strongest predictors of symptomatic VTE. According to Beal et al., high BMI, pancreatic surgery, prolonged operation time, and high transfusion history pose a high‐risk potential in hepato‐pancreato‐biliary surgery.^16^ Though our study did not delve into a detailed examination of VTE risk factors due to the relatively low VTE incidence rate, considering past literature and the occurrence of VTE in the enoxaparin administration group, factors such as DM, splenectomy, and prior surgical history may play an important role in assessing the risk of VTE occurrence during the perioperative phase of malignant tumors in the hepatobiliary‐pancreatic field. Although further case accumulation and examination are required, a more proactive approach to VTE prevention and treatment, including enoxaparin administration for high‐risk groups, seems necessary. Also, in the full analysis set, there were more cases of PTE in the enoxaparin group compared to the control group (two cases vs. one case). Upon further examination of the cases of PTE, it was noted that they had a history of DM and underwent distal pancreatectomy. In this study, there was no significant difference in the proportion of distal pancreatectomy cases between the groups. As mentioned earlier, this study did not investigate the factor of a history of DM. Therefore, this factor may be considered as a potential explanation for the higher number of PTE cases in the enoxaparin group compared to the control group. Further research is needed to investigate the presence of a history of DM and other factors related to the occurrence of PTE and DVT in future studies.

This study has several limitations. While aiming to evaluate the efficacy of perioperative VTE risk reduction in hepatobiliary‐pancreatic malignancies, the majority of cases involved liver resections: 88.2% in the control group and 89.9% in the enoxaparin group. Though this study holds particular significance in assessing the prophylactic effect of enoxaparin administration for perioperative VTE risk in liver resection for malignancies, the sample size for biliary or pancreatic surgery is not sufficient. Therefore, it would be meaningful to consider carrying out clinical trials with a focus on biliary and pancreatic surgery, particularly in the context of future case accumulation. Furthermore, minimally invasive surgery (MIS) has gained prevalence in the hepatobiliary‐pancreatic domain. The possibility of differing VTE incidence rates between conventional open surgery and MIS has been suggested. In this study, randomization was not performed based on surgical approach, highlighting the need for more detailed investigation from the same perspective. Additionally, there were more cases of minor bleeding in the enoxaparin group in both full analysis set and per‐protocol set. While most of cases with minor bleeding improved upon discontinuation of enoxaparin administration, further investigation is needed to assess the risk of bleeding associated with enoxaparin administration, and additional case accumulation and selection are necessary. On the other hand, regarding major bleeding, it was not observed in either the full analysis set or per protocol set for the enoxaparin group, suggesting a potential lack of increased risk of major bleeding associated with enoxaparin treatment.

## 5 CONCLUSION

The perioperative administration of enoxaparin in hepatobiliary‐pancreatic malignancies is feasible and safe. However, further case accumulation and investigation are necessary to assess its potential in reducing the occurrence of VTE.

## AUTHOR CONTRIBUTIONS

Conception and design: S. Kobayashi, T. Noda, and H. Eguchi. Development of the methodology: Y. Takeda, S. Kobayashi, H. Wada, O. Morimoto, A. Tomokuni, J. Shimizu, T. Asaoka, T. Masahiro, T. Noda, Y. Doki, and H. Eguchi. Acquisition of data: G. Shinke, Y. Takeda, Y. Ohmura, S. Kobayashi, H. Wada, O. Morimoto, A. Tomokuni, J. Shimizu, T. Asaoka, T. Masahiro, T. Noda, Y. Doki, and H. Eguchi. Analysis and interpretation of data: G. Shinke, Y. Takeda, Y. Ohmura, S. Kobayashi, H. Wada, O. Morimoto, A. Tomokuni, J. Shimizu, T. Asaoka, T. Masahiro, T. Noda, Y. Doki, and H. Eguchi. Writing, review, and revision of the manuscript: G. Shinke, Y. Takeda, Y. Ohmura, S. Kobayashi, H. Wada, O. Morimoto, A. Tomokuni, J. Shimizu, T. Asaoka, T. Masahiro, T. Noda, Y. Doki, and H. Eguchi.

## FUNDING INFORMATION

The authors have no sources of funding to declare.

## CONFLICT OF INTEREST STATEMENT

Yuichiro Doki is an editorial board member of *Annals of Gastroenterological Surgery*. Other authors declare no conflicts of interest for this article.

## ETHICS STATEMENT

Approval of the research protocol: The Human Ethics Review Committee of each institute approved the study protocol.

Informed Consent: Subjects have provided written informed consent.

Registry and the Registration No. of the study/trial: This study was performed in accordance with the Declarations of Helsinki. Registration number: UMIN000019368.

Animal Studies: N/A.
